# Administrative Data in Cardiovascular Research—A Comparison of Polish National Health Fund and CRAFT Registry Data

**DOI:** 10.3390/ijerph191911964

**Published:** 2022-09-22

**Authors:** Cezary Maciejewski, Krzysztof Ozierański, Mikołaj Basza, Piotr Lodziński, Andrzej Śliwczyński, Leszek Kraj, Maciej Janusz Krajsman, Jefte Prado Paulino, Agata Tymińska, Grzegorz Opolski, Andrzej Cacko, Marcin Grabowski, Paweł Balsam

**Affiliations:** 1 1st Chair and Department of Cardiology, Medical University of Warsaw, 02-091 Warszawa, Poland; 2 Doctoral School, Medical University of Warsaw, 02-091 Warszawa, Poland; 3 Medical University of Silesia in Katowice, 40-055 Katowice, Poland; 4 Satellite Campus in Warsaw, University of Humanities and Economics in Lodz, 90-212 Lodz, Poland; 5 Department of Molecular Biology, Institute of Genetics and Animal Biotechnology, Polish Academy Science, Postępu 36A, 05-552 Magdalenka, Poland; 6 Department of Medical Informatics and Telemedicine, Medical University of Warsaw, 02-091 Warszawa, Poland

**Keywords:** billing data, administrative data, NHF, NLP, cardiology, AF, atrial fibrillation

## Abstract

(1) Background: Administrative data allows for time- and cost-efficient acquisition of large volumes of individual patient data invaluable for evaluation of the prevalence of diseases and clinical outcomes. The aim of the study was to evaluate the accuracy of data collected from the Polish National Health Fund (NHF), from a researcher’s perspective, in regard to a cohort of atrial fibrillation patients. (2) Methods: NHF data regarding atrial fibrillation and common cardiovascular comorbidities was compared with the data collected manually from the individual patients’ health records (IHR) collected in the retrospective CRAFT registry (NCT02987062). (3) Results: Data from the NHF underestimated the proportion of patients with AF (NHF = 83% vs. IHR = 100%) while overestimating the proportion of patients with other cardiovascular comorbidities in the cohort. Significantly higher CHA2DS2VASc (Median, [Q1–Q3]) (NHF: 1, [0–2]; vs. IHR: 1, [0–1]; *p* < 0.001) and HAS-BLED (Median, [Q1–Q3]) (NHF: 4, [2–6] vs. IHR: 3, [2–5]; *p* < 0.001) scores were calculated according to NHF in comparison to IHR data, respectively. (4) Conclusions: Clinical researchers should be aware that significant differences between IHR and billing data in cardiovascular research can be observed which should be acknowledged while drawing conclusions from administrative data-based cohorts. Natural Language Processing of IHR could further increase administrative data quality in the future.

## 1. Introduction

Atrial fibrillation (AF) is a common cardiac arrhythmia affecting 2–4% of adults in the European population [1]. Its increasing prevalence is related to the ageing of modern societies and the presence of other comorbidities (i.e., hypertension, coronary artery disease, heart failure) [1]. Large cohort studies may allow for monitoring of the quality of treatment and patient outcomes in those with AF and possibly lead to new discoveries.

Manual chart review or clinician-driven prospective data collection are regarded as the most accurate methods for clinical research database formation [2]. However, these methods are extremely laborious and time-consuming. They are not feasible for studies that require large cohorts of patients and, therefore, alternatives are being sought.

Administrative (billing) data, most frequently relying on International Classification of Diseases (e.g., ICD-9 and ICD-10) diagnostic codes, are becoming frequently used in observational clinical research. The analysis of ICD codes allows for time- and cost-efficient acquisition of large volumes of individual patient data. The utilization of this data source may in turn allow for the evaluation of real-life clinical outcomes of patients or generation and initial verification of new hypotheses that otherwise could not be tested due to high costs of prospective registry and randomized studies [3]. Administrative data are used extensively for cardiovascular observational clinical studies especially in Northern America and Scandinavian countries due to the availability of large databases [3,4,5]. However, a crucial issue is the reliability of the gathered information. Identification of common cardiovascular diseases in administrative datasets has often shown poor sensitivity and was characterized by a high degree of variability in the past [6,7].

Following the international trends, administrative data is also increasingly being used for clinical research in Poland. In Poland, the NHF provides almost universal healthcare coverage in both inpatient and outpatient settings to its citizens. Since it is the single public health fund of the country, its data makes for a very promising opportunities in clinical research.

The current study aimed to evaluate the accuracy of administrative NHF data from a clinical researcher perspective. NHF data is compared against the data collected manually from the individual patients’ medical documentation in the retrospective CRAFT registry (NCT02987062) [8]. We evaluated the main disease (AF) and several common comorbidities. To the authors’ best knowledge this is the first study of its kind, being based on Polish administrative data and one of the few studies simultaneously evaluating several cardiovascular comorbidities, thus broadening perspectives on the topic.

## 2. Materials and Methods

Due to the retrospective character of the study, the approval of a local ethics committee and patient-provided written informed consent were waived.

### 2.1. Individual Health Record (IHR)—Data Obtained through Manual Chart Review

The current study is based on the cohort of patients collected in the MultiCenter expeRience in AFib patients treated with oral anticoagulation registry (CRAFT NCT02987062). This was a retrospective observational cohort study that included consecutive patients aged ≥18 years, with a diagnosis of AF treated with anticoagulants and hospitalized between 2011–2016 at one academic and one district hospital in Poland. Details about the study design and main results have been reported elsewhere [9]. Case ascertainment of diseases within medical charts was based on: list of discharge diagnoses, hospitalization summary, discharge recommendations and laboratory tests results. Participants with valvular AF were excluded from the analysis due to difficulty in selection of the optimal ICD-10 codes constellation for this clinical diagnosis.

### 2.2. National Health Fund (NHF)—Administrative Data

Unidentified billing data on medical services were acquired from the Polish National Health Fund. NHF provides health care for Polish citizens, with an enrollment rate of approximately 94% of the Polish population. NHF gathers data about medical services that it finances, e.g., exact dates of provision, voivodeship (similar to province), setting (emergency department, inpatient and outpatient), primary diagnosis (ICD-10 code- each medical service has 1 primary diagnosis assigned), procedures (ICD-9 code). The primary diagnosis dictates the need for treatment and/or diagnostic tests and is mainly responsible for the use of resources.

We established the list of ICD-10 codes that served as proxies for actual diagnoses evaluated in the CRAFT study. The set of ICD-10 codes was identified through agreement of two physicians after the analysis of the ICD-10 textbook and is presented in the supplementary material (Supplementary Table S1). These codes were utilized in order to obtain clinical characteristics of patients according to NHF data at the time of the CRAFT study data collection. We analyzed the entire medical history (all types of medical services) registered in the NHF database before and until 30 days after discharge from the hospital at the time of inclusion in the CRAFT registry. We allowed for this 30-day period after hospitalization in order to register additional ICD-10 codes that were likely related to the hospitalization. We decided that such an approach might allow for detection of additional diagnoses acquired from referrals to outpatient care recommended by the treating physician at discharge from the hospital (and thus increase the sensitivity of disease detection). The total number of medical services with assigned ICD-10 codes for this cohort of 3338 patients was 565,521.

### 2.3. CHA2DS2VASc and HASBLED Scores

With regard to CHA2DS2VASc score [10], proxies for all components of the scale could be identified in NHF data.

HASBLED scale was calculated only for data available for evaluation using ICD-10 codes therefore waiving: uncontrolled hypertension, labile prothrombin time, concomitant use of non-steroid anti-inflammatory drugs and antiplatelets. This resulted in a maximum possible score of 6 out of 9 total points in the scale (in this regard, the same calculation method was utilized for IHR and NHF data). Additionally, one component of the NHF-based HASBLED score, “history of severe bleeding”, was analyzed only in the emergency department or inpatient registered billing data in order to identify clinically significant bleeding. Renal disease and liver disease was considered positive according to NHF if any of the selected ICD-10 codes for the respective diseases was present.

### 2.4. Statistical Analysis

In all analyses, IHR data was treated as a reference for NHF data. The results were presented as medians and quartiles for continuous variables and as frequencies and percentages for categorical and ordinal variables. The frequencies of the categorical and ordinal variables were compared with Fisher’s exact test and continuous variables by Mann–Whitney U test respectively. *p* value below 0.05 was considered significant for all tests. All tests were two-tailed.

Sensitivity, specificity, PPV (Positive Predictive Value) and NPV (Negative Predictive Value) were calculated for NHF identified diseases.

Inter-rater reliability between IHR and NHF data with regard to reported diagnoses was analyzed through calculation of Cohen’s Kappa coefficient. The results of this statistic should be analyzed as follows: ≤0 as indicating no agreement between analyzed data sources; 0.01–0.20 as none to slight, 0.21–0.40 as fair, 0.41–0.60 as moderate, 0.61–0.80 as substantial, and 0.81–1.00 as almost perfect agreement.

Statistical analyses and all calculations were performed using R software, version 3.6.2 (R Foundation for Statistical Computing, Vienna, Austria).

## 3. Results

### 3.1. Study Population

**Figure 1:**
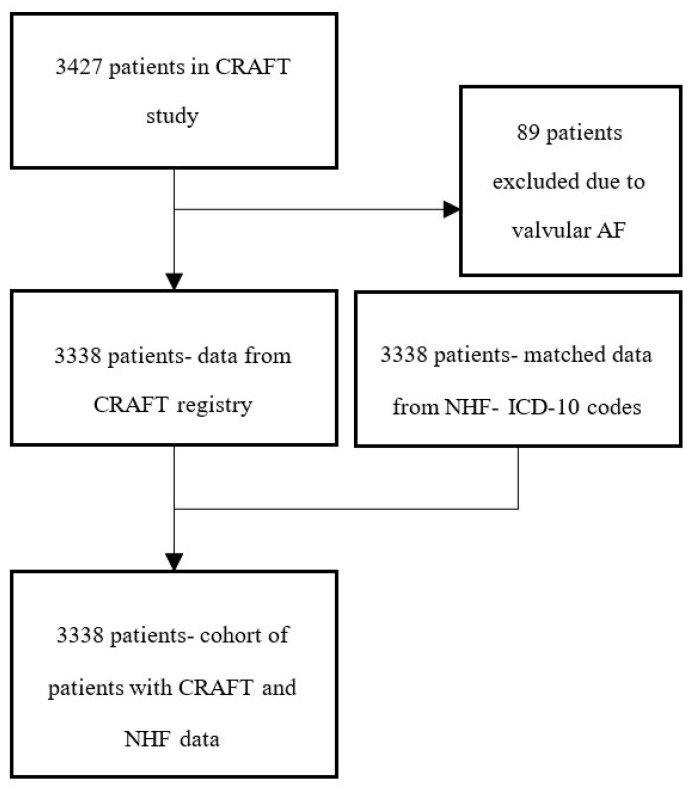
Patient-flow diagram in the study.

The final dataset for analysis consisted of records of 3338 patients with both manually collected data from IHR and NHF data collected through a set of ICD-10 codes detection (Supplementary Table S1). A patient flow diagram is presented in Figure 1. The entire available database consisted of 3427 patients; 89 patients were excluded from the current analysis due to valvular AF diagnosis. For the remaining 3338 records, successful matching with administrative data was achieved.

### 3.2. IHR vs. NHF Data

Table 1 presents the comparison of the entire cohort between IHR and NHF with respective statistics. IHR data is treated as a reference. In all diagnoses there were significant differences present between IHR and NHF. In general, NHF data had a propensity to identify more patients with the respective diagnosis than IHR. NHF underestimated the proportion of AF (all the patients in the CRAFT registry had confirmed diagnosis of AF) and CKD in the cohort while overestimating the proportion of patients with other conditions. The highest sensitivity and PPV was present for CHA2DS2VASc for guideline- recommended anticoagulation use (class I recommendation for anticoagulation use in AF-2 points for men and 3 points for woman in CHA2DS2VASc score), hypertension and atherosclerosis. The highest specificity was present for liver disease, smoking, severe bleeding and alcohol consumption. The highest NPV was present for alcohol, CKD for HASBLED and HASBLED ≥ 3. In the analysis of inter-rater reliability (Cohens kappa) for most diagnoses, there was a fair and slight agreement between IHR and NHF data. The highest agreement was noted for diabetes and prediabetic conditions (moderate agreement) and the lowest for smoking history (none to slight agreement).

**Figure 2:**
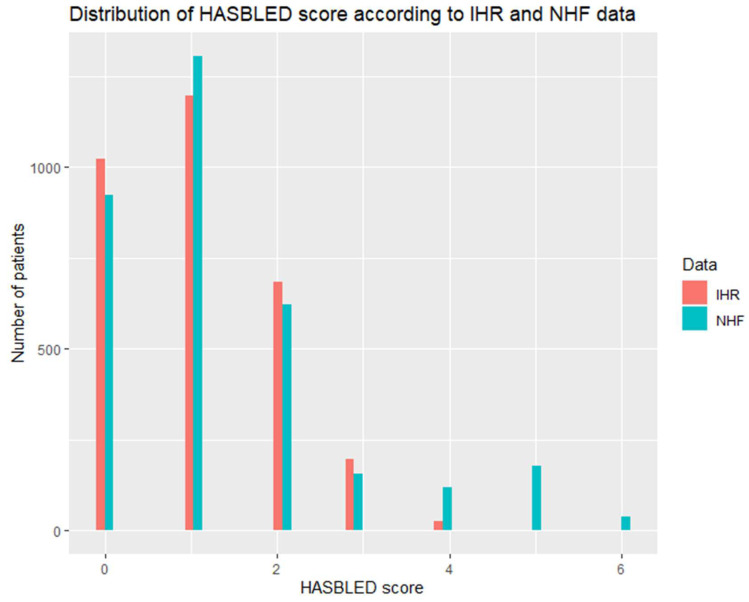
Distribution of HASBLED score within the cohort according to IHR and NHF data.

**Figure 3:**
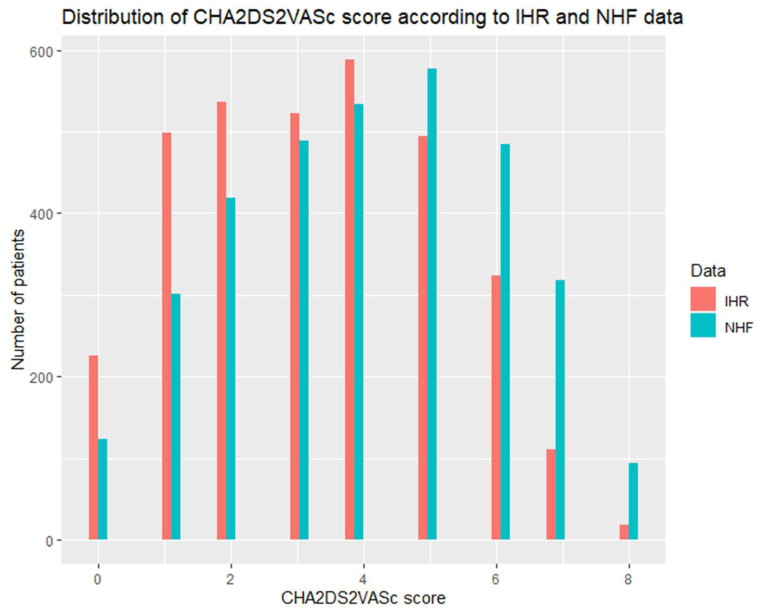
Distribution of CHA2DS2VASc score within the cohort according to IHR and NHF data.

CHA2DS2VASc and HASBLED scores data are presented in several ways which might be confusing to the reader at first but provide in-depth insight to the data gathered in the study. In general, CHA2DS2VASc and HASBLED scores were significantly higher according to NHF data (Table 2). Table 1 presents a comparison of the percentage of patients fulfilling the criteria for class I recommendation for anticoagulation use in AF (2 points for men and 3 points for woman in CHA2DS2VASc score) [1]. NHF data identified more patients fulfilling the criterion. Similarly, NHF identified more patients with HASBLED of ≥ 3 which is referred to as a population with a high risk of bleeding in the current guidelines [1]. Figure 2 and Figure 3 show the comparison of distribution of CHA2DS2VASc and HASBLED scores between IHR and NHF. For both scales, a clear tendency towards higher scoring in the NHF databank is visible. Additionally, confusion matrices for CHA2DS2VASc and HASBLED scores are available in the supplementary materials providing in depth insight to the data (Supplementary Tables S2–S5).

NHF data had high sensitivity, moderate PPV, low specificity and low NPV with regard to identification of patients with class I anticoagulation recommendation. However, this result should be analyzed with caution as the criterion for CRAFT registry inclusion was AF and current anticoagulation intake; therefore, the major proportion of patients fulfill the indication for chronic anticoagulation due to AF with only a minority having transient indication. This biases the results towards high PPV and low NPV [11].

With regard to identification of the population at high risk of bleeding (HASBLED ≥ 3 points), NHF had low sensitivity, low PPV, moderate specificity and high NPV. In this regard, the results are more informative, as the design of CRAFT registry as such did not greatly influence the distribution of HASBLED components.

Table 3 presents the AF patients cohort according to NHF data in comparison with the IHR based cohort. This analysis is presented in order to inform the readers performing clinical studies how utilization of solely administrative data could influence the shape of the final AF patients’ cohort. The NHF based cohort is smaller due to 572 unidentified AF cases. Statistically significant differences in regards to all analyzed comorbidities are present and the NHF based cohort appears to be more burdened in general.

## 4. Discussion

In general, NHF data tended to have relatively low PPV values, indicating that often there are patients classified as having a certain disease according to billing data who do not have it according to individual health records. At the same time, NHF data in most cases showed reasonable NPV. Therefore, if no information about a certain disease is present in the administrative data, then it is very likely the individual does not have it. The results of the performed study suggest that diagnoses collected in administrative data may carry a varying degree of both under-coding (not registering ICD-10 code when the disease is present, which decreases sensitivity and NPV) and over-coding (registering ICD-10 code when the disease is absent, which decreases specificity and PPV). From the authors’ own everyday clinical experience one of the situations when undercoding may arise is when an important diagnosis is not expressed as a corresponding ICD-10 code (even though it is clearly stated in the discharge summary). On the other hand, overcoding may, for instance, emerge when the patient with suspicion of a certain disease is referred to the specialist for evaluation with ICD-10 code of the suspected disease already assigned (not the code expressing suspicion of the disease as should be done)—in such a scenario, even if the disease is excluded after diagnostic process, the ICD-10 diagnostic code will be at least once registered in the patient’s billing data. Both the above-mentioned situations may pose a challenge in designing an observational clinical study based on billing data. The results provided suggest that every clinical condition is defined in the billing data by its own individual qualities and this has to be acknowledged by researchers performing clinical studies utilizing administrative data.

Multiple other studies have evaluated billing data for different cardiovascular diseases and reported conflicting results. In the subsequent paragraphs we will summarize key results of these studies to provide a broader perspective on the topic and present our results in light of other publications. Most of them evaluated the administrative data for one or only a few diseases at a time. For clarity, key statistics in the discussion will be always presented in the following order: sensitivity (Se), specificity (Sp), positive predictive value (PPV), negative predictive value (NPV). Importantly, not all studies provided all of the above-mentioned metrics.

Yao et al. [12] performed a systematic review evaluating the accuracy of AF detection in administrative data that included 24 studies utilizing data from different countries. The pooled estimates were: Se: 80% (95% CI 72–86%); Sp: 98% (96–99%); PPV 88% (82–94%); NPV 97% (94–99%). Authors concluded that billing data may fail to identify a significant proportion of patients with AF and this may affect estimates of quality of care and prognosis in this patient group. In another study on AF, authors evaluated the impact of different strategies for automatic detection of AF in both administrative and electronic health records in USA [13]. Administrative data based on AF diagnosis had a sensitivity of 88%. The utilization of the model employing Natural Language Processing (NLP) of IHR (textual data in electronic health records) detected an additional 22% of patients with AF. The highest predictional value of the presence of AF were achieved for models using a combination of ICD-10 and NLP of individual patients’ electronic health records (EHR). Through a series of simulations with different cohort determination methods (administrative data only, NLP of EHR only, combination of administrative and NLP data with trained machine learning models) it was found that the final number of AF patients that would be included in the cohort could vary by an absolute range of up to 30%, depending on which method had been used for cohort detection. The sensitivity of AF detection based on administrative data in our study corresponds to that of the abovementioned studies.

In a meta-analysis of 11 studies evaluating the accuracy of heart failure diagnosis in administrative databases the calculated statistics were as follows: pooled sensitivity 75.3% (95% CI: 74.7–75.9); pooled specificity 96.8% (95% CI: 96.8–96.9); PPV ≥87% in the majority of studies [14]. Our cohort displayed similar sensitivity and somewhat lower specificity and PPV.

So L. et al. [15] evaluated the accuracy of identification of acute myocardial infarction and comorbidities within Canadian administrative dataset with the following results: HF (Se: 80.0%; Sp: 97.8%; PPV: 93.6%; NPV: 92.5%), CKD (Se: 72.2%; Sp: 98.3%; PPV: 81.3%; NPV: 97.2%); diabetes (Se: 66.7%; Sp: 98.9%; PPV: 83.3%; NPV: 97.2%). Xu et al. [16] performed an analysis of different algorithms including relying solely on ICD-10 codes from Canadian dataset in detection of HF (Se: 60.0; Sp: 99.1; PPV: 93.2; NPV 92.1). The researchers also tested algorithms relying on keyword searches, text mining, and a machine learning model trained with combination of text mining and ICD-10 codes, achieving the highest accuracy with the latter. In another study performed on a Canadian cohort, the following statistics were found: CKD (PPV: 94.3%); COPD (Se: 60.9%; Sp: 94.5%; PPV 84.3%); diabetes (Se: 85.9%; Sp: 97.2%; PPV: 96.2%); liver disease (Se: 13.3%; Sp: 99.7%; PPV: 25.0%); CKD (Se: 50.2%; Sp: 96.6%; PPV: 71.6%). The authors concluded that the prevalence of heart failure and common comorbidities were underestimated in administrative data. Our study differed in that we utilized not only ICD-10 codes related to hospitalization but also to outpatient visits which could influence the final results. The results display similar limitations (e.g., very comparable, poor results in terms of liver disease detection in comparison to our cohort).

The detection of bleeding episodes was evaluated in the study by Joos et al. [17] in USA administrative data. In this study, authors examined charts of patients treated with anticoagulants who were admitted to the hospital. Presence of bleeding related ICD-10 code in any diagnosis position was deemed as positive for bleeding. The results were as follows: Se: 91.4%; Sp: 90.2%; PPV: 52.5%; NPV: 98.9%. The authors concluded that due to a high number of false positive rates, ICD-10 codes should not be used for identifying bleeding complications without confirmatory chart review.

Chang et al. [18] performed an analysis of the association of ICD-9 billing codes with actual diagnoses in the Paul Coverdell National Acute Stroke Program (PCNASP) database and demonstrated high agreement between this registry and administrative data; the Cohen’s kappa coefficient was above 0.9 (almost perfect agreement). The calculated Cohen’s kappa in our study was significantly lower—0.35 (fair agreement). It should be noted, however, that data was analyzed only for hospitals participating in the PCNASP program, therefore likely putting a higher emphasis on the entire care process (including coding accuracy) than non-participating institutions. This may limit the possibility of generalization of these results to the entire healthcare system.

Our study is unique in that we evaluated the main disease (atrial fibrillation) and several common cardiovascular comorbidities simultaneously which was rarely done in prior publications. This offers the reader a comprehensive view on the topic. The results of our study show that characteristics of patients based solely on administrative data may differ from that collected from IHR through manual chart review. Our results and prior evidence cited in the discussion give an important insight into the use of administrative data in cardiovascular research. The final cohort of AF patients based on NHF would be significantly smaller and in general more burdened than that obtained through IHR analysis. These observations are consistent with prior evidence available from other healthcare systems. Clinical researchers should therefore be aware of potential limitations of studies that utilize billing data as the only source of information for diseases and outcome determinations.

The authors believe that administrative data, despite limitations, is an invaluable tool in the arsenal of methodologies that a clinical researcher can utilize in cardiovascular studies. Continuous progress should be made to augment the accuracy of administrative data in order to further expand its use in cardiovascular research. Text mining and natural language processing (NLP) leverages the unstructured narrative from routine care and is another option for identifying patient cohorts. Future efforts should probably focus on increasing the usage of NLP of textual IHR data and artificial intelligence algorithms on top of the analyzed textual data and administrative data which—as pointed out in the discussion—have so far provided promising results. Such efforts may increase the data quality in cardiovascular studies [16]. Such developments may be carried out shortly on a large scale in Poland since the NHF is transitioning to a universal, central electronic documentation platform that is responsible for gathering various types of textual data (discharge summaries, discharge recommendations) and laboratory tests results related to a patient’s given healthcare encounter. This abundance of data, if used efficiently through a combination of ICD codes analysis augmented with text processing and laboratory examinations, may provide researchers with the tools needed for efficient conduct of large, real-world data analysis based observational studies grounded in superior data quality. Notably, availability of the mentioned data types could have substantially minimized most of the limitations we faced during conduct of the present study.

### Limitations

In this study, the manual chart review of patients’ health records was a reference. This is a limitation since the CRAFT registry was collected retrospectively from the patients’ health records and therefore carries inherent limitations of such study design, e.g., missing data. Additionally, underappreciation of certain diseases in IHR data is possible, since the information regarding certain diseases included in the discharge summary is often based on medical history taken from the patient which may be a subject for recall bias. These two drawbacks of our IHR-reference could falsely decrease specificity and PPV of the billing data. Although we consider our manually reviewed IHR data to be of high quality, the true gold-standard would require prospective data collection with source data regarding disease diagnosis verification which could prevent errors resulting from data loss and recall bias.

Secondly, we utilized only the main-diagnosis ICD-10 code as we did not have access to secondary diagnoses ICD-10 codes gathered by NHF which could also affect our results by decreasing sensitivity and NPV of administrative databank. This drawback may be mitigated by the fact that with a databank as big as the NHF, even if one disease is not coded by one provider, another one will likely introduce the code for it at some point of patient’s disease course and it will eventually become evident. However, it cannot be precluded that utilization of secondary diagnosis codes would bring some degree of improvement in the detection of diagnoses; therefore, future studies should aim for their inclusion.

The third limitation is that the exact HASBLED score could not be calculated due to an inability to design adequate proxies for all of its components in administrative data; some of the factors of the scale were simply omitted (the reason for 6 points maximum) as described in the methods section. The others, namely renal disease, liver disease and alcohol use, are very precisely defined in the HASBLED scale (including laboratory thresholds). This level of detail could not be well reflected with a set of ICD-10 codes and likely leads to overestimation of prevalence. This partially explains why significantly more patients are scored for renal disease, liver disease and alcohol use in administrative claims; thus serves as a possible justification for the overall higher HASBLED scores according to NHF than IHR data.

Lastly, our study cannot be broadly generalized; the results are applicable only to NHF data as administrative data accuracy can vary widely, depending not only upon the country and region but also the time period. In Poland, continuous efforts are made by the NHF to increase coding accuracy. As this study concerns data up until the year 2016, many things could have changed and likely for better since then.

## 5. Conclusions

In the present study we evaluated for the first time the accuracy of administrative NHF data for detection of common cardiovascular comorbidities. Although billing databanks remain an invaluable data source, clinical researchers should be aware of their potential limitations as described in the study. Future efforts should probably focus on implementation of Natural Language Processing of individual health records which could further increase data accuracy.

## Figures and Tables

**Table 1:** Comparison between IHR and NHF data. IHR data is treated as reference.

Condition	IHR N, % (CI)	NHF N, % (CI)	*p*-Value	Sensitivity	Specificity	PPV	NPV	Accuracy	Cohen’s Kappa *
AF	3338 100% (99.9–100.0)	2766 83% (81.5–84.1)	**<0.001**	0.83	-	-	-	-	-
Severe bleeding	255/3336 7.6% (6.8–8.6)	409 12.3% (11.2–13.4)	**<0.001**	0.24	0.89	0.15	0.93	0.84	0.01
Alcohol consumption	33/3330 1% (0.7–1.4)	377 11.3% (10.3–12.4)	**<0.001**	0.48	0.89	0.04	0.99	0.88	0.06
CKD for HASBLED	94/3325 2.8% (2.3–3.4)	557 16.7% (15.5–18)	**<0.001**	0.56	0.84	0.1	0.99	0.84	0.12
CKD	706/3325 21.2% (19.9–22.7)	557 16.7% (15.5–18)	**<0.001**	0.34	0.88	0.43	0.83	0.76	0.23
Liver disease	80/3148 2.5% (2–3.2)	346 10.4% (9.4–11.4)	**<0.001**	0.15	0.90	0.04	0.98	0.88	0.02
HF	1207/3333 36.2% (34.6–37.9)	1823 54.6% (52.9–56.3)	**<0.001**	0.82	0.61	0.55	0.86	0.69	0.39
Hypertension	2389/3334 71.7% (70.1–73.2)	2768 82.9% (81.2–84.2)	**<0.001**	0.89	0.32	0.77	0.53	0.73	0.23
Diabetes and prediabetic conditions	874/3325 26.3% (25–27.8)	1108 33.2% (32–34.8)	**<0.001**	0.79	0.83	0.63	0.92	0.82	0.58
Stroke/TIA/ other thromboembolic events	430/3330 12.9% (11.8–14.1)	850 25.5% (24–27)	**<0.001**	0.69	0.81	0.35	0.95	0.79	0.35
Atherosclerosis	1430 42.8% (41.2–44.5)	2390 71.6% (70–73.1)	**<0.001**	0.88	0.40	0.53	0.83	0.61	0.26
CAD	1386 41.5% (40–43.2)	2298 68.9% (67.3–70.4)	**<0.001**	0.86	0.43	0.52	0.81	0.61	0.26
COPD	293/3333 8.8% (7.8–9.8)	735 22% (20.6–23.5)	**<0.001**	0.71	0.83	0.28	0.97	0.82	0.32
Smoking history	175/3328 5.3% (4.6–6.1)	326 9.8% (8.8–10.8)	**<0.001**	0.10	0.90	0.06	0.95	0.86	0.004
HASBLED ≥ 3	86/3124 2.8% (2.2–3.4)	487 14.6% (13.4–15.8)	**<0.001**	0.38	0.86	0.07	0.98	0.85	0.08
CHA2DS2VASc for recommended anticoagulation	2390/3316 72.1% (70.5–73.6)	2816 84.4% (83.1–85.6)	**<0.001**	0.96	0.44	0.82	0.79	0.81	0.46

CI—confidence interval; AF—Atrial Fibrillation; CKD for HASBLED—dialysis, transplant, Cr > 2.26 mg/dL or >200 µmol/L; CKD—any evidence of chronic kidney disease; HF—heart failure; TIA—transient ischemic attack; CAD—coronary artery disease; COPD—chronic obstructive pulmonary disease. * Cohen’s kappa statistic interpretation: ≤0 as indicating no agreement between analyzed data sources; 0.01–0.20 as none to slight, 0.21–0.40 as fair, 0.41–0.60 as moderate, 0.61–0.80 as substantial, and 0.81–1.00 as almost perfect agreement. Numbers after slash “/” refer to available number of cases if there is missing data. Statistically significant differences are marked as **bolded**.

**Table 2:** Comparison of HASBLED and CHA2DS2VASc scores in IHR and NHF data.

	IHR Median [Q1–Q3]	NHF Median [Q1–Q3]	*p* Value
HASBLED	1 [0–1] *3124*	1 [0–2]	**<0.001**
CHA2DS2VASc	3 [2–5] *3316*	4 [2–6]	**<0.001**

Q1, Q3—1st and 3rd quartile; Numbers after slash “/” refer to available number of cases if there is missing data. Statistically significant differences are marked as **bolded**. Numbers in *italics* refer to available number of cases with complete data for respective scale calculation.

**Table 3:** Summary statistics of the cohort of AF patients in IHR and NHF datasets.

Condition	IHR N = 3338 N, % (CI)	NHF N = 2766 N, % (CI)	*p*-Value
Severe bleeding	255/3336 7.6% (6.8–8.6)	378 13.7% (12.4–15)	**<0.001**
Alcohol consumption	33/3330 1% (0.7–1.4)	360 13% (11.8–14.3)	**<0.001**
CKD for HASBLED	94/3325 2.8% (2.3–3.4)	524 18.9% (17.5–20.5)	**<0.001**
CKD	706/3325 21.2% (19.9–22.7)	524 18.9% (17.5–20.5)	**0.03**
Liver disease	80/3148 2.5% (2–3.2)	343 12.4% (11.2–13.7)	**<0.001**
HF	1207/3333 36.2% (34.6–37.9)	1576 57% (55.1–59)	**<0.001**
Hypertension	2389/3334 71.7% (70.1–73.2)	2408 87% (85.8–88)	**<0.001**
Diabetes	874/3325 26.3% (25–27.8)	951 34.4% (32.6–36.2)	**<0.001**
Stroke/TIA/other thromboembolic events	430/3330 12.9% (11.8–14.1)	758 27.4% (25.8–29.1)	**<0.001**
Atherosclerosis	1430 42.8% (41.2–44.5)	2066 74.7% (73–76.3)	**<0.001**
COPD	293/3333 8.8% (7.8–9.8)	671 24.3% (22.7–25.9)	**<0.001**
CAD	1386 41.5% (40–43.2)	1998 72.2% (70.5–73.9)	**<0.001**
Smoking history	175/3328 5.3% (4.6–6.1)	323 11.7% (10.5–13)	**<0.001**
HASBLED ≥ 3	86/3124 2.8% (2.2–3.4)	470 17% (15.6–18.4)	**<0.001**
HASBLED, median [Q1–Q3]	1 [0–1]	1 [0–2]	**<0.001**
CHA2DS2VASc for recommended anticoagulation	2390/3316 72.1% (70.5–73.6)	2364 85.5% (84.1–86.7)	**<0.001**
CHA2DS2VASc, median [Q1–Q3]	3.000 [2–5]	4.0 [3–6]	**<0.001**

CI—confidence interval; Q1,Q3—1st and 3rd quartile; CKD for HASBLED—dialysis, transplant, Cr > 2.26 mg/dL or >200 µmol/L; CKD—any evidence of chronic kidney disease; HF—heart failure; TIA—transient ischemic attack; COPD—chronic obstructive pulmonary disease; CAD—coronary artery disease; Numbers after slash “/” refer to available number of cases if there is missing data. Statistically significant differences are marked as **bolded**.

## Data Availability

Not applicable.

## Supplementary Materials

The following supporting information can be downloaded at: https://www.mdpi.com/article/10.3390/ijerph191911964/s1, Table S1: ICD-10 codes of clinical diagnoses analyzed in the study; Table S2: Confusion matrix for HASBLED score; Table S3: Confusion matrix for HASBLED score according to ≥3 points cutoff—high risk of bleeding according to 2020 ESC AF guidelines; Table S4: Confusion matrix for CHA2DS2VASc score; Table S5: Confusion matrix for CHA2DS2VASc score according to ≥2 points for men and ≥3 points for woman cutoff—class I recommendation for chronic anticoagulation in atrial fibrillation according to 2020 ESC AF guidelines.

## References

[1] Hindricks G., Potpara T., Dagres N., Arbelo E., Bax J.J., Blomström-Lundqvist C., Boriani G., Castella M., Dan G.A., Dilaveris P.E. (2021). 2020 ESC Guidelines for the diagnosis and management of atrial fibrillation developed in collaboration with the European Association for Cardio-Thoracic Surgery (EACTS). Eur. Heart J..

[2] Lee D.S., Donovan L., Austin P.C., Gong Y., Liu P.P., Rouleau J.L., Tu J.V. (2005). Comparison of coding of heart failure and comorbidities in administrative and clinical data for use in outcomes research. Med. Care.

[3] Lip G.Y.H., Keshishian A., Li X., Hamilton M., Masseria C., Gupta K., Luo X., Mardekian J., Friend K., Nadkarni A. (2018). Effectiveness and Safety of Oral Anticoagulants Among Nonvalvular Atrial Fibrillation Patients. Stroke.

[4] Ray W.A., Chung C.P., Murray K.T., Smalley W.E., Daugherty J.R., Dupont W.D., Stein C.M. (2018). Association of Oral Anticoagulants and Proton Pump Inhibitor Cotherapy With Hospitalization for Upper Gastrointestinal Tract Bleeding. JAMA.

[5] Schmidt M., Schmidt S.A.J., Sandegaard J.L., Ehrenstein V., Pedersen L., Sørensen H.T. (2015). The Danish National Patient Registry: A review of content, data quality, and research potential. Clin. Epidemiol..

[6] Quach S., Blais C., Quan H. (2010). Administrative data have high variation in validity for recording heart failure. Can. J. Cardiol..

[7] Kaspar M., Fette G., Güder G., Seidlmayer L., Ertl M., Dietrich G., Greger H., Puppe F., Störk S. (2018). Underestimated prevalence of heart failure in hospital inpatients: A comparison of ICD codes and discharge letter information. Clin. Res. Cardiol..

[8] Balsam P., Tymińska A., Ozierański K., Zaleska M., Żukowska K., Szepietowska K., Maciejewski K., Peller M., Grabowski M., Lodziński P. (2020). Randomized controlled clinical trials versus real-life atrial fibrillation patients treated with oral anticoagulants. Do we treat the same patients?. Cardiol. J..

[9] Balsam P., Ozieranski K., Tyminska A., Zukowska K., Zaleska M., Szepietowska K., Maciejewski K., Peller M., Grabowski M., Lodzinski P. (2018). Comparison of clinical characteristics of real-life atrial fibrillation patients treated with vitamin K antagonists, dabigatran, and rivaroxaban: Results from the CRAFT study. Kardiol. Pol..

[10] Lip G.Y., Nieuwlaat R., Pisters R., Lane D.A., Crijns H.J. (2010). Refining clinical risk stratification for predicting stroke and thromboembolism in atrial fibrillation using a novel risk factor-based approach: The euro heart survey on atrial fibrillation. Chest.

[11] Parikh R., Mathai A., Parikh S., Chandra Sekhar G., Thomas R. (2008). Understanding and using sensitivity, specificity and predictive values. Indian J. Ophthalmol..

[12] Yao R.J.R., Andrade J.G., Deyell M.W., Jackson H., McAlister F.A., Hawkins N.M. (2019). Sensitivity, specificity, positive and negative predictive values of identifying atrial fibrillation using administrative data: A systematic review and meta-analysis. Clin. Epidemiol..

[13] Shah R.U., Mukherjee R., Zhang Y., Jones A.E., Springer J., Hackett I., Steinberg B.A., Lloyd-Jones D.M., Chapman W.W. (2020). Impact of Different Electronic Cohort Definitions to Identify Patients With Atrial Fibrillation From the Electronic Medical Record. J. Am. Heart Assoc..

[14] McCormick N., Lacaille D., Bhole V., Avina-Zubieta J.A. (2014). Validity of heart failure diagnoses in administrative databases: A systematic review and meta-analysis. PLoS ONE.

[15] So L., Evans D., Quan H. (2006). ICD-10 coding algorithms for defining comorbidities of acute myocardial infarction. BMC Health Serv. Res..

[16] Xu Y., Lee S., Martin E., D’Souza A.G., Doktorchik C.T.A., Jiang J., Lee S., Eastwood C.A., Fine N., Hemmelgarn B. (2020). Enhancing ICD-Code-Based Case Definition for Heart Failure Using Electronic Medical Record Data. J. Card. Fail..

[17] Joos C., Lawrence K., Jones A.E., Johnson S.A., Witt D.M. (2019). Accuracy of ICD-10 codes for identifying hospitalizations for acute anticoagulation therapy-related bleeding events. Thromb. Res..

[18] Chang T.E., Lichtman J.H., Goldstein L.B., George M.G. (2016). Accuracy of ICD-9-CM Codes by Hospital Characteristics and Stroke Severity: Paul Coverdell National Acute Stroke Program. J. Am. Heart Assoc..

