# Supplementary material for: Administrative Data in Cardiovascular Research—A Comparison of Polish National Health Fund and CRAFT Registry Data
Source: Int J Environ Res Public Health. 2022 Sep 22;19(19):11964. doi: 10.3390/ijerph191911964 (PMC9565600; doi:10.3390/ijerph191911964)
Supplement: Supplementary file 1 [file ijerph-19-11964-s001.zip › ijerph-1860356-supplementary.pdf]

Supplementary Table S1. ICD-10 codes of clinical diagnoses analyzed in the study

Clinical diagnosis	ICD-10 (International Classification of Diseases) codes
AF	I48
Severe bleeding (intracranial, gastrointestinal, respiratory, renal/urinary tract, ocular, retroperitoneal or pericardial bleeding episodes)	D50.0 R04.0 R31.0 R31.9 I71.3 J94.2<br>H05.2 H21.0 H31.3 H31.4 H35.6<br>H43.1 H44.8 H47.0 R58 M25.0<br>M79.8 N83.7 N89.7 N92.0 N92.1<br>N92.5 N93.0 N93.8 N93.9 O71.7<br>R04.1 R04.2 R04.89 R04.9<br>D62 D68.3 D69.8 D69.9 H11.3<br>H45.0 H92.2 I23.0 I31.2 S26.0 S27.1<br>T79.2 N02 R31 N42.1 N92.4 T81.0<br>Y60 R58 R04 N02.0 N02.1<br>N02.2 N02.3 N02.4 N02.5 N02.6<br>N02.7 N02.8 N02.9 N42.1 N92.3<br>N95.0 R04.8 N93 N85.7 R23.3 I85.0<br>K22.1 K25.4 K26.4 K27.0 K29.0<br>K29.7 K31.8 K92.0 K57.3 K57.9<br>K62.5 K92.1 K92.2 K25.0 K25.2<br>K25.6 K26.0 K26.2 K26.6 K27.2<br>K27.4 K27.6 K28.0 K28.2 K28.4<br>K28.6 K29.2 K29.3 K29.4 K29.5<br>K29.6 K29.8 K55.2 K57.0 K57.1<br>K57.2 K57.4 K57.5 K57.8 I98.3<br>K22.6 K66.1 K76.2 K62.6 K63.3 I84.4<br>I62.0 I62.9 I60 I60.0 I60.1 I60.2<br>I60.3 I60.4 I60.5 I60.6 I60.7 I60.8<br>I60.9 I61 I61.0 I61.1 I61.2 I61.3<br>I61.4 I61.5 I61.6 I61.8 I61.9 I62.1<br>I62 S06.4 S06.5 S06.6
Alcohol consumption (alcohol-related medical services)	I42.6 F10 F10.0 F10.1 F10.2 F10.3<br>F10.7 F10.8 F10.9 F10.3 F10.4 F10.5<br>F10.6 F10.7 F10.8 F10.9 F15.7<br>G31.2 G62.1 G72.1 I42.6 K29.2 K70<br>K70.0 K70.1 K70.2 K70.3 K70.4<br>K70.9 K86.0 T51
CKD (chronic glomerulonephritis, chronic tubulointerstitial nephropathy, diabetic, and hypertensive nephropathy)	N99.0 I12.0 N03.6 N18 N18.0<br>N18.8 N18.9 G63.8 I32.8 N18 N19
Liver disease (chronic liver disease, liver cirrhosis, hepatitis)	K70 K70.9 K71.0 K71.1 K71.2<br>K71.3 K71.4 K71.5 K71.6 K71.7<br>K71.8 K71.9 K75.9 K76.5 K76.9<br>K77.0 K70.4 K71.1 K72 K72.1<br>K72.9 K70.2 K71.7 K74 K74.0<br>K74.2
HF (including cardiomyopathies)	I50 I50.0 I50.1 I50.9 I42.0 I25.5<br>I42 I42.4 I42.9 O90.3 I42.0 I42.1<br>I42.2 I42.3 I42.6 I42.7 I42.8 I42.9<br>I43.8 I13.0 I13.2 I43 I43.0 I43.1
Hypertension	I10 I11 I11.0 I11.9 I15 I15.0<br>I15.1 I15.2 I15.8 I15.9 O11
Diabetes and prediabetic conditions	E10 E11 N08.3 E10.0 E10.1 E10.2<br>E10.3 E10.3 E10.4 E10.5 E10.6 E10.7<br>E10.8 E10.9 E10.0 E11.1 E11.2 E11.3<br>E11.3 E11.4 E11.5 E11.6 E11.7 E11.8

	E11.9 E13 E14 E12 E13 E14<br>E10.0 E10.1 E10.2 E10.3 E10.4 E10.5<br>E10.6 E11.0 E11.1 E11.2 E11.3 E11.4<br>E11.5 E11.6 E12.0 E12.1 E12.2 E12.3<br>E12.4 E12.5 E12.6 E13.0 E13.1 E13.2<br>E13.3 E13.4 E13.5 E13.6 E14.0 E14.1<br>E14.2 E14.3 E14.4 E14.5 E14.6 E15<br>G59.0 G63.2 G73.0 G99.0 H28.0<br>H36.0 I79.2 M14.2 M14.6 R40<br>R73.0 T38.3 Y42.3
Stroke/TIA/ other thromboembolic events	I63 I63.0 I63.1 I63.2 I63.3 I63.4<br>I63.5 I63.6 I63.8 I63.9 I64 I69.3<br>I69.4 G45 G45.3 G45.4 G45.6<br>G45.9 I74 I74.0 I74.1 I74.2 I74.3<br>I74.4 I74.5 I74.8 I74.9 I63.0 I63.0<br>I63.1 I63.2 I65.1 I65.2 I63.3 I63.4<br>I63.5 I66.9 K55.0 I63.0 I63.1 I63.2<br>I65.9 N28.0 I65.0 K55.0 I63 G45<br>I65 K55.0 I74 M31.1 N28.0<br>H34.1 G45.8 G45.9 I63.0 I63.1 I63.2<br>I63.3 I63.4 I63.5 I63.8 I63.9 I67.8<br>I67.9 I64 I74.0 I74.1 I74.2 I74.3<br>I74.4 I74.5 I74.8 I74.9
Atherosclerosis (myocardial infarction, peripheral artery disease and other atherosclerotic conditions)	I70 I70.0 I70.1 I70.2 I70.8 I70.9<br>I67.2 I25.1 K55.1 I27.0 Z95.1 Z95.5<br>I21 I21.0 I21.0 I21.1 I21.2 I21.3<br>I21.4 I21.9 I20 I20.0 I20.8 I20.9<br>I20.1 I25 I25.0 I25.1 I25.2 I25.6<br>I25.5 I25.0 I25.1 I67.2 I70 I70.0<br>I70.1 I70.2 I70.8 I70.9 I70.2 I70.8<br>I70.9 I70.1 I70.2 I70.8 I70.9
CAD	Z95.1 Z95.5 I21 I21.0 I21.0 I21.1<br>I21.2 I21.3 I21.4 I21.9 I20 I20.0<br>I20.8 I20.9 I20.1 I25 I25.0 I25.1<br>I25.2 I25.6 I25.5
COPD	J44.0 J44.1 J44.8 J44.9 J44
Smoking history	F17.2 T65.2 F17 F17.0 F17.1 F17.2<br>F17.3 F17.4 F17.5 F17.6 F17.7 F17.8<br>F17.9 Z71.6 Z72.0 Z58.7

AF- Atrial Fibrillation; CKD- chronic kidney disease; HF- heart failure; TIA- transient ischemic attack; CAD-coronary artery disease; COPD- chronic obstructive pulmonary disease.

Supplementary Table S2. Confusion matrix for HASBLED score

-	NHF	0	1	2	3	4	5	6
IHR	-							
0		834	145	18	1	82	8	0
1		57	879	334	70	17	117	19
2		4	156	181	59	9	34	13
3		1	19	26	17	6	4	3
4		0	5	2	1	1	1	0
5		0	0	0	0	0	0	0
6		0	0	0	0	0	0	0

Supplementary Table S3. Confusion matrix for HASBLED score according to >=3 points cutoff - high risk of bleeding according to 2020 ESC AF guidelines

-	NHF	<3	>=3
IHR	-		
<3		2608	429
>=3		53	33

Supplementary Table S4. Confusion matrix for CHA2DS2VASc score

-	NHF	0	1	2	3	4	5	6	7	8	9
IHR	-										
0		69	73	49	14	2	18	0	0	0	0
1		35	146	148	95	27	37	10	0	0	0
2		13	55	136	166	91	39	30	6	0	0
3		4	14	41	127	168	99	39	30	0	0
4		1	8	17	42	168	178	111	54	9	0
5		0	2	15	25	59	146	151	76	20	0
6		0	1	6	9	13	48	119	91	36	0
7		0	0	0	7	3	8	21	53	19	0
8		0	0	0	2	0	1	0	5	10	0
9		0	0	0	0	0	0	0	0	0	0

Supplementary Table S5. Confusion matrix for CHA2DS2VASc score according to >=2 points for men and >= 3 points for woman cutoff– class I recommendation for chronic anticoagulation in atrial fibrillation according to 2020 ESC AF guidelines

Chronic anticoagulation recommended	-	NHF	No	Yes
	IHR	-		
	No		410	516
	Yes		107	2282
